# Spatial information from the odour environment in mammalian olfaction

**DOI:** 10.1007/s00441-020-03395-3

**Published:** 2021-01-30

**Authors:** Alina Cristina Marin, Andreas T Schaefer, Tobias Ackels

**Affiliations:** 1 Sensory Circuits and Neurotechnology Laboratory, The Francis Crick Institute, London, UK; 2 Department of Neuroscience, Physiology & Pharmacology, University College London, London, UK

**Keywords:** Spatial information, Odour, Mammalian olfaction, Odour plumes

## Abstract

The sense of smell is an essential modality for many species, in particular nocturnal and crepuscular mammals, to gather information about their environment. Olfactory cues provide information over a large range of distances, allowing behaviours ranging from simple detection and recognition of objects, to tracking trails and navigating using odour plumes from afar. In this review, we discuss the features of the natural olfactory environment and provide a brief overview of how odour information can be sampled and might be represented and processed by the mammalian olfactory system. Finally, we discuss recent behavioural approaches that address how mammals extract spatial information from the environment in three different contexts: odour trail tracking, odour plume tracking and, more general, olfactory-guided navigation. Recent technological developments have seen the spatiotemporal aspect of mammalian olfaction gain significant attention, and we discuss both the promising aspects of rapidly developing paradigms and stimulus control technologies as well as their limitations. We conclude that, while still in its beginnings, research on the odour environment offers an entry point into understanding the mechanisms how mammals extract information about space.

## Introduction

Every organism regardless of biological complexity is constantly exposed to a plethora of different sensory stimuli that they need to parse to obtain information about the nature and location of predator, prey, mates and themselves. For nocturnal and crepuscular animals, such as common laboratory species like rats and mice, the sense of smell is particularly a crucial sense for gaining insight into the external world. When visual cues are limited or even absent, olfactory cues are especially useful, as they provide information over a large range of distances, allowing behaviours from the simple detection and recognition of objects, to tracking and navigating using distant odour plumes. In this review, we will discuss recent work into the central questions of which spatial information animals can extract from the olfactory environment and highlight progress made both in terms of understanding the physics of the olfactory scenery, behavioural experimental approaches and initial work on neural mechanisms underlying these behaviours. We will focus on mammals, in particular mice and rats, whilst only touching upon the rich work performed in invertebrates, where research into olfactory-driven navigation is significantly more advanced, as outlined in a number of excellent reviews (Baker et al. 2018; Cardé and Willis 2008; Vickers 2000).

In the following sections, we will first outline the features of a natural olfactory environment and the type of information present in odour plumes. We will then provide a brief overview of how this information is sampled and might be extracted by the mammalian olfactory system. Finally, we will describe recent developments of different behavioural paradigms aimed to address how mammals extract spatial information from the environment in three specific settings, odour trail tracking, odour plume tracking and, more general, olfactory-guided navigation. While this field is still in its infancy, both experimental paradigms and stimulus control technologies are rapidly developing. This points towards a bright future where experiments investigating how the olfactory environment can inform mammals about space might provide a gateway into a general mechanistic understanding of how the mammalian nervous system extracts spatial information.

## Physical features of the olfactory environment

The olfactory world comprises an enormous variety of odours that rarely occur in isolation. Rather, it is made up of complex olfactory mixtures which vary in composition and concentration of their constituent odour molecules (Mori et al. 1999). This potentially provides a rich picture of the environment that can be harnessed over a large range of animal behaviours, from detecting food sources to complex navigation strategies required to find mates or avoid predators.

**Fig. 1:**
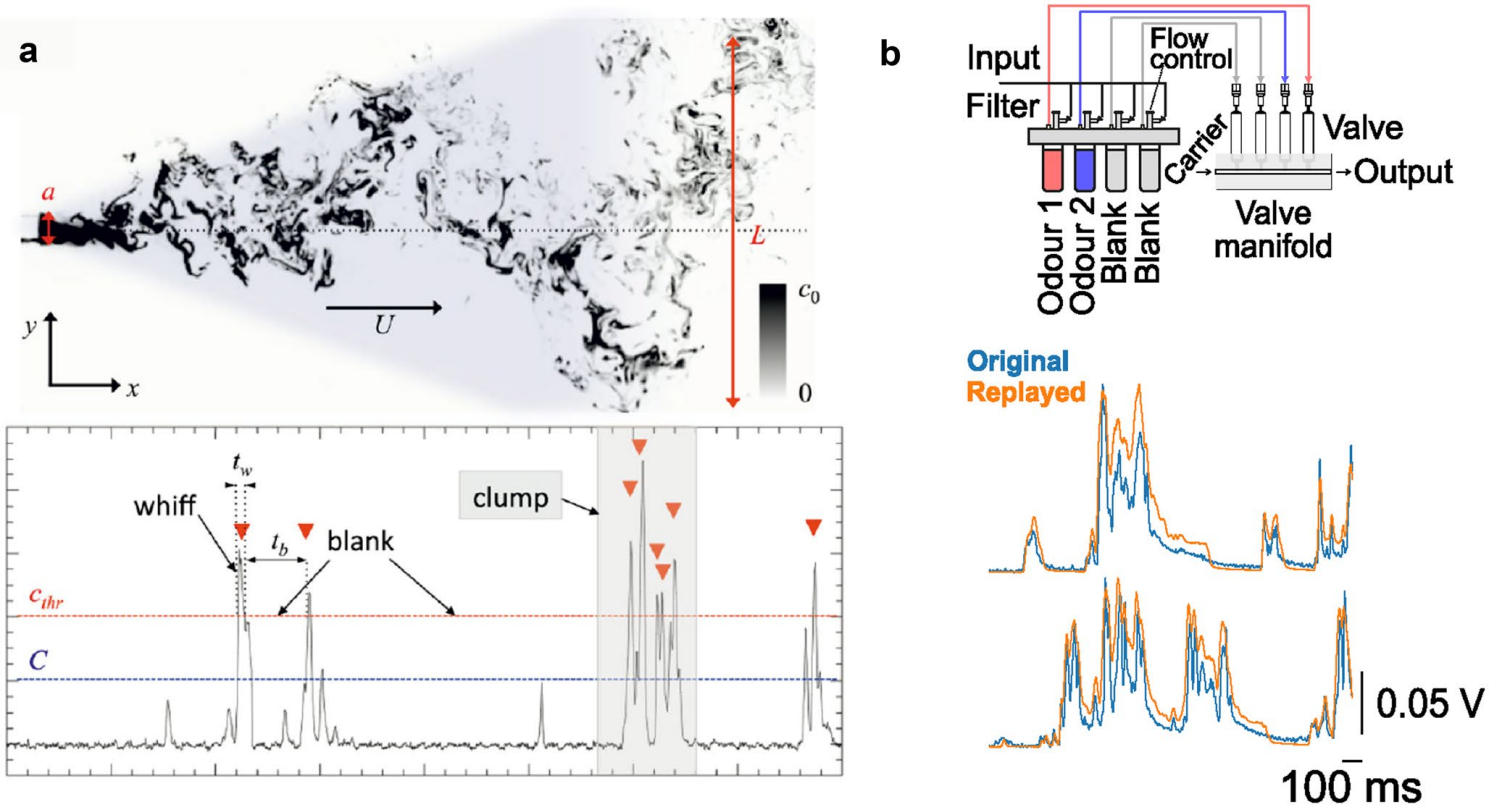
Structure and reproduction of complex odour plumes. **a** *Top*: Two-dimensional section of a turbulent odour plume highlighting its chaotic distribution in water. *Bottom*: Recording of the odour concentration fluctuation over time at a given point in space (from Celani et al. 2014). **b** *Top*: Schematic of a multi-channel high bandwidth odour delivery device (adapted from Erskine et al. 2019). *Bottom*: Two example plume structures as recorded with a photoionization detector (PID) (blue) and replayed with the multi-channel high bandwidth odour delivery device (orange; Marin, Ackels, Dasgupta, Warner, Schaefer, unpublished results)

Importantly, natural odorants are not stationary but are often transported as plumes by complex air movements generated by environmental conditions (Fig. 1a). The turbulent nature of this airflow disturbs any gradients that might form through diffusion and structures the plume in isolated patches of varying concentrations, creating an odour signal that is dynamic in both space and time (Celani et al. 2014; Moore and Crimaldi 2004; Murlis et al. 1992; Mylne and Mason 1991; Shraiman and Siggia 2000). Thus, fluid dynamic conditions play a major role in shaping the spatiotemporal structure of the odour plume and olfactory signals in general. Such spatiotemporal structures of odour plumes have been described in detail for aquatic and air environments, often in the context of studying olfactory navigation in crustaceans or insects (Celani et al. 2014; Justus et al. 2002; Moore and Crimaldi 2004; Murlis 1997; Murlis and Jones 1981; Mylne and Mason 1991). The spatiotemporal structure of odour plumes can be recorded from single locations downstream of an odour source using photoionisation detectors (Justus et al. 2002), proton transfer reaction mass spectrometry (Riffell et al. 2014) or electroantennography (Arn et al. 1975; Schneider 1957). While technically more challenging and with some trade-off in temporal resolution, planar laser-induced fluorescence allows the simultaneous measurement of a large number of spatial locations (Connor et al. 2018; Crimaldi and Koseff 2001). Turbulent plumes have been recorded over longer ranges and in different outdoor environments by employing gas tracers (Mylne and Mason 1991), pheromone plumes (Murlis et al. 2000) and flower scents (Riffell et al. 2014). Laboratory studies have been carried over shorter distances inside tightly controlled wind tunnels (Justus et al. 2002; Vickers et al. 2001; Victor et al. 2019), and computational fluid dynamics simulations can be employed alongside such recordings to investigate information encoded in odour plumes (Boie et al. 2018).

How do animals deal with the complexity of the olfactory environment? Do they adopt more complex strategies to solve behavioural challenges, and if so, which? Extensive research in insects and crustaceans shows that animals are not only capable of navigating in turbulent odour plumes, but they might also use the turbulent aspect of odour plumes to perform odour source localisation. Some animals are thought to use the fluctuations in odour plumes as navigation cues (Keller and Weissburg 2004; Koehl 2001; Mafra-Neto and Cardé 1994), while others use odour-gated anemotaxis, a strategy that combines odour detection with multisensory integration of information about the environment, with the resulting behaviour of upwind movement in the presence of an odour, and casting in the absence of the odour (Kennedy and Marsh 1974; Murlis et al. 1992).

Much less is known in mammals compared with invertebrates. In addition to the complexity of the environment discussed above, a challenge faced by mammalian research is that of access to the stimulus: In insects, olfactory sensory neurons on antenna and maxillary palp are accessible and directly exposed to the odour stimulus, and electroantennogram recordings can provide an estimate of the temporal profile of the odour stimuli reaching the animal (Arn et al. 1975; Schneider 1957). In mammals, odours are sampled with active sniffing, and thereby are sucked through the turbinates of the nasal cavity and need to pass a mucus layer before they reach the olfactory receptor neurons that are experimentally poorly accessible, buried deep in the nasal cavity. Measuring the dynamics of an odour plume at the naris with, e.g., sucking air into a fast photoionization detector (PID), will inevitably substantially perturb the odour stimulus itself. We will discuss in “Sampling of odour information” how sampling behaviours gate olfactory sensory perception and are modulated by both stimulus and context features. Understanding the link between stimulus features, sampling behaviours and navigation is an active field of research (Findley et al. 2020; Jordan et al. 2018a, 2018b). Due to the difficulties in measuring the exact spatiotemporal profile of a turbulent odour plume as it is sampled by an animal, a more recent strategy to investigate the link between complex stimuli and an animal’s response is to employ high-speed odour delivery devices (Fig. 1b) that can deliver temporally complex odour stimuli, including reproduced odour plume structures (Erskine et al. 2019; Raiser et al. 2017).

As airflow turbulence is a result of the environment in which it is created, it has been suggested that the spatiotemporal structure of the plume contains information about the location, distance and composition of odour sources: The temporal structure imposed on odour concentration dynamics by turbulent air flow can aid in performing source separation by identifying chemicals emerging from the same source by their correlated concentration fluctuations (Erskine et al. 2019; Hopfield 1991). Furthermore, as a plume widens in space as it travels, the statistics of concentration fluctuations will also change with increasing distance to the source (Moore and Atema 1991; Murlis et al. 1992, 2000; Vickers et al. 2001; Weissburg et al. 2002). Moreover, several features have been identified to vary reliably with distance to the source, such as height and onset slope of a peak (Moore and Atema 1991), intermittency (Riffell et al. 2014) and average bout count (Schmuker et al. 2016). In these settings, odour molecules are transported across distances of centimetres or even metres, leaving convection rather than diffusion as the dominant force of transport at these length scales. Insects and crustaceans, in particular, have been observed to follow chemical plumes to sources that routinely are tens to hundreds, and even thousands, of body lengths away (Moore and Crimaldi 2004; Weissburg et al. 2002).

## Sampling of odour information

The continuous gathering of information about the olfactory environment is often crucial for animal survival. Odour sampling behaviour is thus the prerequisite for an animal to reliably and quickly assess its ever-changing olfactory surroundings. Across modalities, sensory information is transformed into neural activity in both temporal and spatial dimensions (Panzeri et al. 2017; Smith 2008). In mammalian olfaction, active sampling of the environment has long been known to shape how odours are represented and processed in the brain (Adrian 1942, 1950; Cang and Isaacson 2003; Cury and Uchida 2010; Macrides and Chorover 1972; Margrie and Schaefer 2003; Shusterman et al. 2011). Active sampling presents an essential asset to selectively regulate stimulus intensity and dynamics to ultimately optimise sensory processing (Wachowiak 2011). The invertebrate olfactory system is continuously exposed to air or water as the external medium. Considered as the functional equivalent of vertebrate sniffing (Atema 1985; Schmitt and Ache 1979), active odour sampling behaviour manifests itself for example as wing beating (Chapman et al. 2018) or antennae flicking (Devine and Atema 1982; Reeder and Ache 1980) imposing additional intermittency on the olfactory stimulus, and thus helping to gain more information about the odour location (Huston et al. 2015).

In terrestrial vertebrates, olfaction depends on the rhythmic inhalation of air into the nasal cavity. These discrete sampling events enable an animal to extract sensory information quickly and reliably, a prerequisite for exploring and assessing the environment. While humans even in active olfactory tasks such as trail following sniff slowly at frequencies < 1 Hz (Porter et al. 2007), sniffing behaviour in rodents covers a wide frequency range of 2–12 Hz (Welker 1964) strongly depending on both stimulus and contextual features such as odour novelty (Esquivelzeta Rabell et al. 2017; Verhagen et al. 2007) and attentiveness of the animal (Jordan et al. 2018a, b; Kepecs et al. 2006; Wachowiak 2011; Wesson et al. 2008; Youngentob et al. 1987). Importantly, active sampling and changes in sniff rate profoundly impact on odour representation in the brain (Jordan et al. 2018a, b; Jordan, Kollo, et al. 2018; Parabucki et al. 2019; Verhagen et al. 2007; Wachowiak 2011), possibly optimising odour representation for the task at hand. While individual sniffs are often viewed as the key ‘unit of information’ for mammalian olfaction, there is increasing evidence that a single sniff does not just provide a discrete olfactory snapshot but rather builds a larger picture: Mice can learn to discriminate between light-evoked inputs at the sub-sniff level when the early olfactory system gets stimulated with optogenetics at only 10–20 ms apart (Smear et al. 2011, 2013).

How does odour sampling relate to the bilateral anatomy of the olfactory system? At first, the flow of information is clearly lateralised as both hemispheres do not have any cross-connection until the anterior olfactory nucleus (Bennett 1968; Brunjes et al. 2005). When inhaling air through both nostrils, vertebrates can compare inter-naris odour information (stereo olfaction; Esquivelzeta Rabell et al. 2017; Rajan et al. 2006). In most mammalian species, the nostrils are located relatively close together, which seems at first glance unfavourable in regard to comparing odour information across nostrils (Moulton 1967). However, constant head movements in highly motile species could compensate for the small spacing between nostrils. Rats take independent, bilateral samples of the odour environment when presented with complex odour plumes. This might generate different concentration fluctuation patterns across both nostrils despite their close proximity (Wilson and Sullivan 1999). Experiments in multiple species have shown that the ability to reliably localise odour sources in many cases depends on bilateral odour sampling. Von Bekesy provided strong evidence for directional smelling in humans (Bekesy 1964). Congruently, when occluding one nostril performance to locate the direction of an odour source is drastically reduced (Welge-Lüssen et al. 2014), and even the detection of systematic subtle manipulations in odour concentration is subject to stereo olfaction in humans (Wu et al. 2020). In a functional brain imaging study, unilaterally delivered odours induced nostril-specific neural activity in the primary olfactory cortex that was predictive of behavioural localisation accuracy (Porter et al. 2005). In line with these human studies, naris occlusion reduced odour trail–tracking ability in rats (Khan et al. 2012) and mice (Jones and Urban 2018) and impaired odour direction sensitivity in moles (Catania 2013). Thus, bilateral odour comparison might contribute information about the odour plume source, reminiscent of sound localisation through the detection of interaural time and level differences (Recanzone and Sutter 2011).

Undeniably, sampling odour information is the precondition for any odour-driven behaviour, profoundly modulating odour representation and perception. There is initial evidence that sampling strategies including those involving both nares are particularly important for extracting information about space. For the remainder of this review, we will discuss some recent developments on how to tackle such ‘spatial behaviours’.

## Odour-driven spatial behaviours in mammals

Despite the recognised importance of olfactory cues in rodent spatial orientation, how the olfactory environment is sampled and whether spatiotemporal features of odour plumes are used by animals on a behavioural and neural level remain a largely understudied topic. Some evidence exists from visual navigation studies that also included olfactory stimuli (Lavenex and Schenk 1995, 1997, 1998; Maaswinkel and Whishaw 1999). A more recent focus on tracking and source localisation behaviours is starting to shed a light on the mechanisms involved from an olfactory sampling and processing perspective. Animals trained to navigate to an odour source will use a multitude of strategies to solve this one question, adopting different strategies and flexibly switching between them based on task, environment, learning level and other behavioural parameters. New techniques are being harnessed to link the sampling behaviours of the animal, captured using video-tracking and respiration measurements, to the olfactory information available in the environment, measured using odour sensors (Findley et al. 2020; Liu et al. 2020). However, it remains unknown what information animals can extract from more complex olfactory stimuli such as odour plumes, and how they use this information to aid navigation in complex environments. This gap in research can partly be attributed to experimental constraints in terms of stimulus control, as well as an incomplete, ever-evolving understanding of complex, turbulent olfactory environments.

Below, we will summarise such tracking, source localisation and general olfaction-guided navigation experiments and explore what these experiments tell us about the role of odour information in questions about space.

### Odour trail tracking

**Fig. 2:**
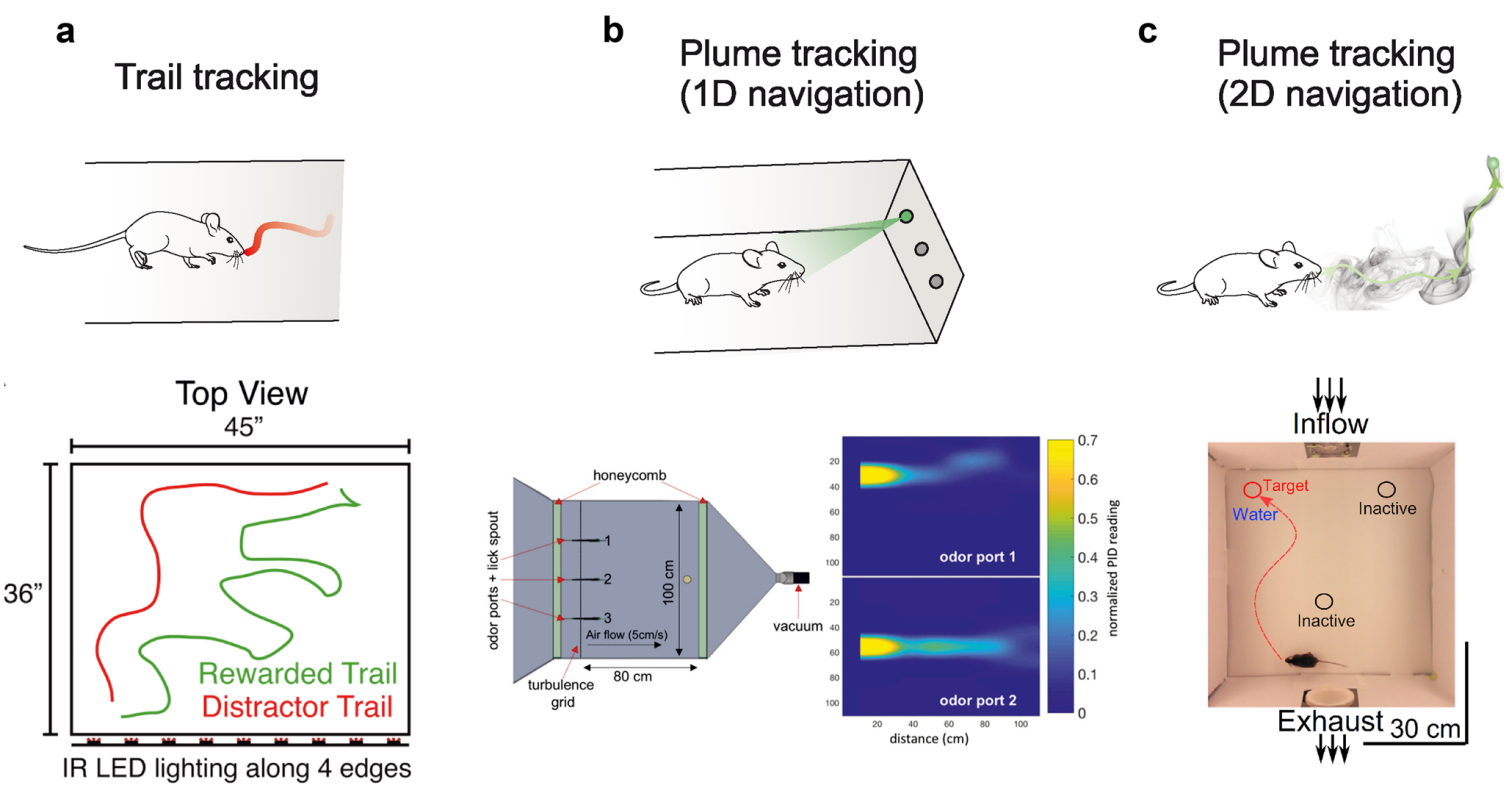
Illustration of experimental setups to study different aspects of odour-driven behaviour. **a** *Top*: Schematic of odour trail tracking behaviour. *Bottom*: Schematic of an arena to record mouse odour trail tracking behaviour by high-resolution video through a transparent floor. The mouse is placed at one end of the trail and tasked to track it along its length (from Jones and Urban 2018). **b** *Top*: Schematic of one-dimensional plume tracking behaviour. *Bottom left*: Schematic of a behavioural chamber with non-turbulent chaotic airflow characteristics to record one-dimensional odour plume tracking behaviour. The animal is rewarded after successfully navigating towards the port releasing odour. *Bottom right*: Time averaged and normalised PID recordings of odour from port 1 and 2 across the flow chamber (from Gumaste et al. 2020). **c** *Top*: Schematic of two-dimensional odour plume tracking behaviour. *Bottom*: Diagram of an arena to study two-dimensional olfactory-guided plume tracking behaviour under turbulent airflow conditions. The animal receives a water reward after successful navigation to the activated odour source (adapted from Gire et al. 2016)

As an example of odour tracking is following a scent trail on the ground (surface-borne cues), formed when odour molecules are deposited on a surface (Fig. 2a). The use of such odour trails by mammals has long been described in field observations. Rodents such as wood mice show a preference to follow trails they had previously laid during exploration (Jamon 1994), and the scent marking of paths, for example with urine, is a common behaviour employed by animals (Arakawa et al. 2008) that has been proposed to allow them to orient themselves within their home range (Benhamou 1989).

In the laboratory, a trail following task would involve moving along the trail and keeping contact with it, until the source is reached. The trail could be drawn on a surface in an open field (Jones and Urban 2018; Porter et al. 2007; Wallace et al. 2002) or on paper spooled through a treadmill (Khan et al. 2012; Mathis et al. 2018). These experiments showed that rats (Khan et al. 2012; Wallace et al. 2002), mice (Jones and Urban 2018) and even humans (Porter et al. 2007) are able to perform this task and accurately track the odour trail. Moreover, rats can use animal-generated odour trails to reach a rewarded location from different starting points in an open-field arena, in the absence of visual cues (Lavenex and Schenk 1998), or even to solve a water-escape “working memory” task (Means et al. 1992). As the task is primarily about following a trail as an animal moves along it, an animal could solve the task using the concentration gradient formed around the trail. Such a strategy would involve inter-naris comparisons (stereo olfaction) and inter-sniff comparisons, combined with scanning movements across the path, similar to casting movements in insects. This is indeed the case: animals scan their nose across the trail, widening the scan path when the nose diverges from the trail. The tracking animals structure their behaviour to increase sniff rate while scanning, using comparisons between consecutive sniffs to follow the trail and inter-naris comparisons to increase their accuracy (Jones and Urban 2018; Khan et al. 2012; Porter et al. 2007). Tracking performance also improves with training, as human subjects show decreased deviation from the track and increased tracking velocity over multiple days of training (Porter et al. 2007).

In addition to following a trail to the source, an animal in the wild would also have to determine the direction of the trail, providing an added layer of complexity. The ability to detect the direction of a trail is crucial for animals looking for prey, and also for tracking dogs. Trained tracking dogs employ a 3-stage process to locate the source of an odour track, involving a searching phase, a deciding phase and a tracking phase (Thesen et al. 1993). The decision has been suggested to be based on concentration comparisons between a small number of adjacent (consecutive) footprints (Hepper and Wells 2005; Steen and Wilsson 1990). Interestingly, a recent study in dogs has found that environmental changes such as humidity and air temperature influence the animal’s sampling behaviour. The dogs moved more slowly and sampled odours closer to the ground under hot and dry conditions, while lower temperatures and higher humidity allowed for more rapid movement while sampling air-borne odours (Jinn et al. 2020). While behaviour and psychophysics along trail tracking in mammals has thus seen encouraging advances over the last years, dissection of neural representation of odour trails and the neural mechanisms behind trail following are only just about to become the target of investigation.

Trail tracking can be particularly useful for animals to track themselves and other animals, and thus to build more complex representations of space. But, an olfactory trail is only a small subset of the olfactory stimuli an animal is faced with in its natural habitat and might need to locate. Animals, for example, may want to orientate themselves relative to distant odour sources, avoid distant predators (without having to follow their trails) or simply search for stationary, often buried objects such as food (Howard et al. 1968), which have not left a trail. In this latter case, while also ‘tracking’ an odour stimulus, they would have to track an odour *plume* to its source to locate this type of object.

### Odour plume tracking

As discussed in the previous sections, airborne olfactory cues are more complex than surface-borne odour trails on multiple counts: (1) the concentration gradients between the inside and the outside of the plume/trail are steeper in odour trails, (2) the concentration gradient along the length of the trail is uniform, whereas for a plume, it fluctuates dramatically at high frequencies and (3) a trail is fixed in space while an odour plume is dynamic, changing shape and position depending on airflow turbulence. Localising the source of an odour plume is thus a different and possibly more complex challenge for the olfactory system. Tracking such airborne plumes, however, is a frequently observed behaviour in many species, including mammals such as dogs (Jinn et al. 2020; Hepper and Wells 2005; Jacobs 2012); it is less well studied in natural ethological behaviours for rodents (Howard et al. 1968; Jacobs 2012).

The complexity of the challenge has also given rise to difficulties in setting up laboratory tasks (Fig. 2b, c) that address the different features of the olfactory environment with appropriate controls. Difficulties can also be attributed to technical challenges in producing and measuring naturalistic olfactory stimuli, further amplified by a lack of intuitive understanding of olfactory environments by the human experimenter, in contrast with the relative ease of designing visual or auditory experiments. In this situation, different promising experimental setups have been developed to simplify certain aspects of the olfactory environment by reducing the number of odours, precisely releasing odours into the airstream and regulating the airflow and thus the turbulence of the odour plume. Initial experiments aimed to control the airflow such that even chemical gradients are formed (Bhattacharyya and Singh Bhalla 2015; Catania 2013). One study in such a gradient showed that in this setting, moles combine the use of bilateral cues with serial sampling to guide navigation to an odour source and offered insights into the relative contribution of each strategy during different stages of search behaviour, with bilateral cues becoming more important in the steeper odour gradients found in proximity to the source (Catania 2013). In a source localisation task using airborne fluctuating plumes, mice could efficiently find the odour source, and their tracking behaviour in the vicinity of the source was consistent with a gradient-based algorithm (Gire et al. 2016). However, as the mice become more experienced with the particular task and reward locations, their strategy shifted to a memory-based, systematic foraging strategy. This is consistent with a finding from a different odour localisation task where rats were trained to run towards on odour source in a multi-choice olfactory arena. Here, rats did not cast and sample different positions in the near-laminar air flow. Instead, they adopted a strategy to move directly towards one target and, if this was incorrect, serially sampled all possible target positions (Bhattacharyya and Singh Bhalla 2015). Experiments that randomly vary odour source location across trials (Jackson et al. 2020; Liu et al. 2020) suggest that animals rely on sensory cues if target locations are less predictable. In a task that discouraged serial-sampling of targets by terminating trials when the mouse approached an unrewarded source, mice could successfully localise the source of an odour plume over a range of airflow conditions (Gumaste et al. 2020). Analysis of behavioural trajectories suggested that mice shifted between different search strategies depending on the complexity of the olfactory environment. The same study also investigated the performance of search robots employing simple inter-naris and temporal models of tropotaxis and klinotaxis to perform the task in the same environment as the mice. Here, they found that robots were equally successful in locating odour sources in environments with low plume complexity. Their performance, however, dropped relative to experimental mice when the complexity of the olfactory environment was increased. Olfactory search robots have numerous applications in localising odour sources in challenging environments or in automation, and their development is an active field of research (Chen and Huang, 2019).

Overall, there are only a few studies to date to have investigated how mammals use olfaction to locate odour sources, and the overarching theme is that mammals employ a range of strategies to perform this task even with increasing complexity in the environment (Bhattacharyya and Singh Bhalla 2015; Catania 2013; Findley et al. 2020; Gire et al. 2016; Gumaste et al. 2020; Jackson et al. 2020; Liu et al. 2020). In turn, investigations into the neural representations and mechanisms of extracting this spatial information are only beginning, possibly aided by the development of effective virtual reality setups (Baker et al. 2018; Fischler et al. 2019; Mathis et al. 2018; Radvansky and Dombeck 2018).

Interestingly, animal behaviour changes systematically with distance to the source, as measured by speed and orientation towards the source (Liu et al. 2020). Findley et al. trained mice to navigate to an odour source in a known environment with a small number of possible targets (Findley et al 2020). Using machine-learning methods to parse behavioural trajectories suggested that navigation motifs fell into two classes consistent with investigation and approach states. Moreover, while sniffing and head movements were tightly synchronised, stereo olfaction was not required for successful navigation (Findley et al. 2020). Consistent with a sensory-informed straight-to-source run, mice can and often do make a decision early in their run (Findley et al. 2020; Gire et al. 2016; Gumaste et al. 2020).

The fact that animals can make these decisions further away from the source suggests that they have built associations between the olfactory stimulus and the rewarded locations, so olfactory cues are integrated into the cognitive spatial map animals use to navigate. This would also allow for more complex behaviours than source localisation to be informed by olfactory cues, which is very likely in animals relying on olfaction for many of their behaviours.

### Olfactory-guided navigation

One key limitation of animal studies is the reliance on appropriate task design for the question asked. This is particularly challenging in complex environments with few predictions of how an animal might behave, as the task employed needs to guide the animal to perform the targeted behaviour, and prevent alternative strategies being exploited, unless they are part of the question. Task design was one challenge behind studies of plume-tracking navigation, due to animals primarily choosing the fastest strategy to get the reward—which in laboratory environments with limited complexity is not necessarily following odour concentration gradients or plumes (Bhattacharyya and Singh Bhalla 2015; Gire et al. 2016). Moreover, the type of strategy and sensory information used by the animal may depend on the type of environment it lives in and in which it performs such searches, as well as environmental conditions at the time of the search (Jinn et al. 2020). With this in mind, one has to consider that experiments performed in a simplified laboratory arena are a great tool to study what an animal can do and chooses to do when faced with a particular task and environment, but also need to be interpreted as simplified versions of a natural environment (Fig. 2c). Great progress has been made in designing tasks closer to the natural ethology: using plumes and turbulent airflow (Findley et al. 2020; Gire et al. 2016; Gumaste et al. 2020; Liu et al., 2020), probing mice with temporally complex stimuli and reproduced odour plumes (Erskine et al. 2019; Fig. 1b), olfactory virtual reality setups (Baker et al. 2018; Fischler et al. 2019; Radvansky and Dombeck 2018), potential for plume-following from a head-mounted odour sensor (Tariq et al. 2019) and wireless devices that could allow for more flexible navigation paradigms. However, we are yet to move beyond the fundamental constraints of using a source localisation task, which is a significant limiting factor in assessing what spatial information animals are able to get from odour plumes.

Experiments in humans could help with this constraint, as one can ask more direct and abstract questions. One such study found that human participants are able to define a location in space and return to that location using odours alone (Jacobs et al. 2015). This result indeed suggests the use of spatial memory to create odour-informed maps of the environment. These maps could be based on the formation of odour “neighbourhoods” anchored to locations in space, demarcated by odour mixing ratios and concentration gradients (Jacobs 2012). In another study, participants were shown to be able to mentally navigate a two-dimensional olfactory space, similar to that described by Jacobs (2012), and this navigation was associated with hexagonal grid-like neural representations in prefrontal and entorhinal cortices (Bao et al. 2019).

Another line of evidence supporting a role of olfactory cues in building a cognitive map of space comes from studies primarily investigating visual navigation, where often underappreciated odour cues such as the scent marks produced by the animal have been suggested to play a role in building spatial representations (Lebedev et al. 2018; Lebedev and Ossadtchi 2018). Consistent with a more central role of the olfactory system in navigation (Jacobs 2012), olfactory bulbectomy in rats severely impairs navigation in rats, even when visual cues are available (van Rijzingen et al. 1995). The importance of odour cues has also been noted in studies on hippocampal representations of space. Place cells established in blind rats are similar to those in sighted rats (Save et al. 1998), and olfaction is a prime candidate for providing the necessary spatial information from distant cues. The presence of odour cues has been shown to have a profound impact on place field formation and stability (Anderson and Jeffery 2003; Muzzio et al. 2009; Save et al. 2000), and also on the preferred direction of head direction cells (Goodridge et al. 1998). A more recent study showed that rats navigating an environment signposted by olfactory cues, in the absence of visual cues, display stable hippocampal place fields that rotate when the odour locations are rotated and remap when odour locations are shuffled (Zhang and Manahan-Vaughan 2015). Recent developments in odour delivery technology have allowed for the construction of olfactory virtual reality setups where mice on a treadmill can be trained to, for example, run between two odour-defined areas, guided only by experimenter-controlled smooth or noisy odour gradients (Baker et al. 2018; Fischler et al. 2019; Radvansky and Dombeck 2018). This behaviour also engages hippocampal “place cells” similar to those reported for visual virtual environments. Spatially selective neurons have also been reported in the piriform cortex of rats performing an odour-cued spatial navigation task, suggesting this area of the brain to be involved in supporting navigational behaviour by associating spatial and olfactory information (Poo et al. 2020).

Rodents commonly used in laboratory studies are crepuscular or nocturnal animals and as such presumably heavily reliant on non-visual cues for navigation. Despite that, the involvement of olfaction in navigation tasks has often been described as a side-note to the visually guided navigation intended in the studies. The aim often is to improve future research by highlighting the need of controls for unintended olfactory stimuli in order to stop animals exploiting odours generated by themselves or conspecifics to complete the navigation task (Means et al. 1992). Indeed, one trail-following study described earlier was performed in this context (Wallace et al. 2002). With the navigation field moving towards virtual reality setups that eliminate the relevance of olfactory cues (Harvey et al. 2009), the need arises for research that specifically addresses olfactory navigation. Conventional navigation tasks using olfactory cues will benefit from employing new technologies in arena design (Findley et al. 2020; Jackson et al. 2020; Liu et al. 2020), video-tracking (Mathis et al. 2018; Wiltschko et al. 2015), respiration measurements (Reisert et al. 2020) and odour data obtained from head-mounted sensors (Tariq et al. 2019) and two-dimensional plume measurements (Connor et al. 2018; Crimaldi and Koseff 2001). This research would further be enriched by olfactory-based virtual reality systems (Radvansky and Dombeck 2018) that can reproduce the richness of turbulent odour environments and also allow for subtle manipulations of the stimuli. This type of system would be ideal for investigations into olfactory space maps that would parallel the visual navigation field. In fact, as the olfactory bulb projects directly to the hippocampal formation, olfaction might provide a promising entry point to dissect the mechanism of how sensory information is employed to shape neural representation of space. Before this can be done, however, we need to further advance our understanding of odour plume dynamics in relation to the spatial environment and employ this knowledge in novel behavioural paradigms including the aforementioned virtual reality ones to expand our description of strategies of olfactory-guided navigation. Combining these with detailed stimulus control, measurements of active sampling, and neurophysiological recordings will pave the way for exploring the neural mechanisms underlying odour-guided navigation.

## Conclusion

The sense of smell provides key information about the environment, especially for crepuscular and nocturnal animals such as mice or rats. Having been understudied for a long time, in recent years, the spatiotemporal aspect of mammalian olfaction has gained significant attention, and considerable progress has been made in understanding the physics of the olfactory scenery, odour-driven behavioural essays, as well as uncovering the first puzzle pieces of underlying neural mechanisms. The need remains for improved and focused behavioural task design, as well as tight control of complex odour stimuli in order to do justice to the richness of spatiotemporal plumes. The compact organisation of the mammalian olfactory system will then allow using spatial information in odours to serve as an entry point to understanding how space is represented in the mammalian brain.
